# Factors associated with adherence to antiretroviral therapy in HIV-infected subjects and the use of indicators to characterize the treatment adhesion profile

**DOI:** 10.1590/1414-431X2023e12738

**Published:** 2023-11-13

**Authors:** E.M. Piegas, M.I. Ziolkowski, R.A. Bittencourt, C.K.C. Malheiros, F.F. Miranda, C.F. Dias, L.P. Mocellin, S.E. Haas

**Affiliations:** 1 Programa de Pós-Graduação em Ciências Farmacêuticas, Laboratório de Farmacologia e Farmacometria, Universidade Federal do Pampa, Uruguaiana, RS, Brasil; 2 Departamento Municipal de Saúde, Uruguaiana, RS, Brasil; 3 Programa de Pós-Graduação em Bioquímica, Universidade Federal do Pampa, Uruguaiana, RS, Brasil; 4 Laboratório de Farmacologia e Farmacometria, Universidade Federal do Pampa, Uruguaiana, RS, Brasil; 5 Curso de Medicina, Universidade Federal do Pampa, Uruguaiana, RS, Brasil

**Keywords:** HIV, cART adherence, Antiretroviral therapy, Pharmacy refill, Adhesion

## Abstract

At present, there is no gold standard to assess patient adherence to combination antiretroviral therapy (cART). Therefore, this study aimed to characterize the epidemiological profile, delineate adherence indicators, and identify factors associated with adherence and delays in obtaining medication in patients registered at the Specialized Assistance Service in HIV/AIDS in Brazil. This is a descriptive study based on secondary data obtained from official databases of the Brazilian Ministry of Health. Adherence and delay were measured by the frequency of cART medication acquisition in 24 months, and a multivariate linear regression model was developed to identify the factors associated with non-adherence and delays. In 50.2% of the subjects, the viral load remained undetectable throughout the study period. Only 12.4% of patients were fully adherent to cART. Regarding indicators, a value of 0.83 was found for adherence, 0.09 for delay in days, and 0.21 for the number of times the patient was late to obtain the medication. The multivariate analysis showed that males, age between 20 and 59 years, having not changed the cART, and the presence of ≥1000 HIV RNA copies/mL were predictive factors for adherence and delays (P≤0.01). We demonstrated that monitoring cART medication distribution is possible using health indicators, and identifying the factors associated with poor adherence to cART helps characterize patients at higher risks of unsuccessful therapy.

## Introduction

The continued increase in human immunodeficiency virus (HIV) infection in the population remains a serious global health problem (1). Despite numerous advances, including the increased use of combination antiretroviral therapy (cART), which has led to a reduction in new HIV infections, dealing with this illness remains a challenge because it is a chronic disease that has no cure. People living with HIV and AIDS (PLWHA) need to be clinically monitored throughout their lives (1,2). An estimated 37.9 million people are infected with HIV worldwide, with 23.3 million people having access to cART. Moreover, AIDS-related illnesses have caused about 32 million deaths since the epidemic began in 1980. In 2018, 1.7 million new cases of HIV infection were reported (3).

In Brazil, 43,941 new HIV cases and 37,791 AIDS cases were reported in 2017, totaling, from 1980 to June 2019, almost one million detected AIDS cases, which is a detection rate of 17.8 and a mortality rate of 4.4/100,000 population (4). The state of Rio Grande do Sul (RS) has a generalized epidemic that requires inter-federative actions to combat HIV and the fifth highest HIV-related detection (27.2/100,000 population) and mortality rates (11.1/100,000 population) in Brazil, which is above the national average (4). In addition, RS had the second highest AIDS detection and mortality rates in the last five years (4).

Regarding Brazilian cities with at least 100,000 inhabitants and AIDS detection rates, four out of ten are in RS. Uruguaiana ranks 52nd nationally (30.2/100,000 population) and has a mortality rate almost four times higher than the national average (19.6/100,000 population) (4). The city borders Argentina and Uruguay and has the largest dry port in Latin America, contributing to the large influx of people into the city (4,5). Border regions are a source of concern for HIV transmission due to significant population movements and are representing critical points for marginalization, sexual exploitation, and drug trafficking (6). Additionally, Uruguaiana has a specialized service for cART provision. Due to its high infection rate, the city is one of the 15 cities considered a priority in RS regarding actions against infection (4).

As a global health emergency, governments, non-governmental organizations, and health services have begun implementing strategies to increase cART adherence and coverage in developing countries (2). Among the plans of action to combat the AIDS epidemic in Brazil, medication distribution policies by the Ministry of Health (MH) since 1996 and the “Test and Treat” policy in 2013 have provided antiretroviral drugs free of charge to anyone diagnosed with HIV, regardless of TCD_4_ lymphocyte counts (7). Disease diagnosis and treatment are the main factors in reducing mortality (8). However, adherence to cART is difficult because the treatment involves several chronically administered drugs that may lead to adverse effects and/or drug-food interactions (9). Additional challenges include establishing bonds with healthcare professionals, accessing health services and information, clinical and laboratory follow-up, adapting to new habits, sharing stigma-related decisions, and successful treatment adherence (10).

Research has shown that cART non-adherence rates in Brazil may vary from 18 to 74.3% (11,12). Hence, it is crucial to identify the gaps in continuous care, such as therapy failure due to lack of efficacy, poor drug adherence, and acquired resistance to cART (2), which are necessary intervention opportunities for this population. Considering the relevance of the HIV infection scenario in Uruguaiana and the consequences of non-adherence to cART, this study aimed to characterize the epidemiological profile, delineate adherence indicators, and identify factors associated with adhesion and delays in cART medication acquisition in patients registered with the Specialized Assistance Service in HIV/AIDS of Uruguaiana.

## Material and Methods

### Ethical considerations

All experimental protocols were in accordance with the Declaration of Helsinki. The researchers signed a confidentiality agreement to guarantee the confidentiality of patient data. Each subject was assigned an identification number, and the analyses were performed considering aggregate data rather than individual data. This study evaluated secondary data and was based on the analysis of databases (see “Study design, settings, and participants”). The informed consent form was waived for the research subjects. All protocols were approved by the Research Ethics Committee of the Federal University of Pampa (UNIPAMPA) (CAAE No. 92602618.3.0000.5323). The specialized health service of Uruguaiana signed an informed consent to conduct the study.

### Study design, settings, and participants

This was a descriptive study based on secondary data and was conducted from January 2017 to December 2018. No drug shortage occurred during this period. Information was collected directly from the federal government health information systems to which only public health services have access. Professionals from the Specialized Assistance Service on HIV/AIDS in Uruguaiana (Brazil) were collaborators in this study, and the consent of the referred service was given, which allowed access to the data. The datasets generated and/or analyzed during the current study were not publicly available but were made available to the corresponding author upon reasonable request. The health service registered 1,344 patients receiving clinical and drug treatment. Survey participants were required to meet the following criteria: be a PLWHA registered in the Uruguaiana specialized health service, be over 12 years old (because at this age the patient can manage their treatment) (13), and have a history of cART use of at least 24 months. Patients who died, were transferred to other cities, underwent treatment for less than 24 months at the beginning of the collection, or were late in acquiring cART medication at least once during this period were excluded.

### Data collection

Sociodemographic information, therapeutic regimens, and cART medication acquisition dates were collected directly from the Logistic Medication Control System (SICLOM). Data of follow-up laboratory tests (CD4+ and CD8+ cell counts; HIV-1 RNA viral loads) were obtained directly from the Brazilian National CD4+/CD8+ T Lymphocyte Count and Viral Load Network Laboratory Test Control System (SISCEL).

### cART adherence measures

Treatment adherence was measured by the frequency of cART medication acquisition in 24 months, regardless of their occurrence, and categorized into the following groups according to the Ministry of Health and the Clinical Protocols and Therapeutic Guidelines (2018) (8,14): 1) Adherents: patients who obtained the cART medication 24 times; 2) Minimum effective adherence: patients who obtained their cART medication between 20 and 23 times; 3) Low adherence: patients who obtained their cART medication between 16 and 19 times; 4) Non-adherent: patients who obtained their cART treatment 15 times or less.

### Total delay of cART medication acquisition

Delay in seeking treatment was also verified using the last 24 medication acquisitions via SICLOM. According to legislative requirements, cART is provided for 30 days of treatment (10). A delay in cART dispensation was considered to be more than 34 days for each month. A binary indicator with “0” and “1” values was also constructed for each patient to classify the number of delays. The value “1” corresponds to the delay in acquiring the medication on the scheduled date, while the value “0” corresponds to no delay in attaining medication.

### cART adherence and delay indicators

Epidemiological indicators included adherence, total number of delayed days, and how often the patient was late in seeking treatment during the study period. The mean value of the indicators was obtained using Equations 1, 2, and 3.

Adhesion Indicator:

$$\frac{Number\;of\;months\;the\;patient\;obtained\;all\;prescribed\;cART}{24\;months}$$ Eq. 1

Days Delayed Indicator:

$$\frac{Sum\;of\;days\;overdue\;during\;survey\;time}{730\;days}$$ Eq. 2

Number of Delays Indicator:

$$\frac{Sum\;of\;all\;delayed\;time\;during\;the\;survey}{24\;months}$$ Eq. 3

### Statistical analysis

A descriptive statistical analysis was performed to identify the frequency distribution of categorical and numerical variables. Spearman's correlation was used between indicators and HIV-1 RNA viral load. The correlations were interpreted according to the following correlation coefficient classification: <0.4 (weak correlation), ≥0.4 to <0.5 (moderate correlation), and ≥0.5 (strong correlation). A multivariate linear regression model was then developed to identify predictors related to non-adherence and treatment delay. Statistical significance was considered at 5% (P≤0.05) in all analyses. The statistical analysis was performed using the SPSS software (version 17.0, IBM, USA).

## Results

### Study participants

**Figure 1:**
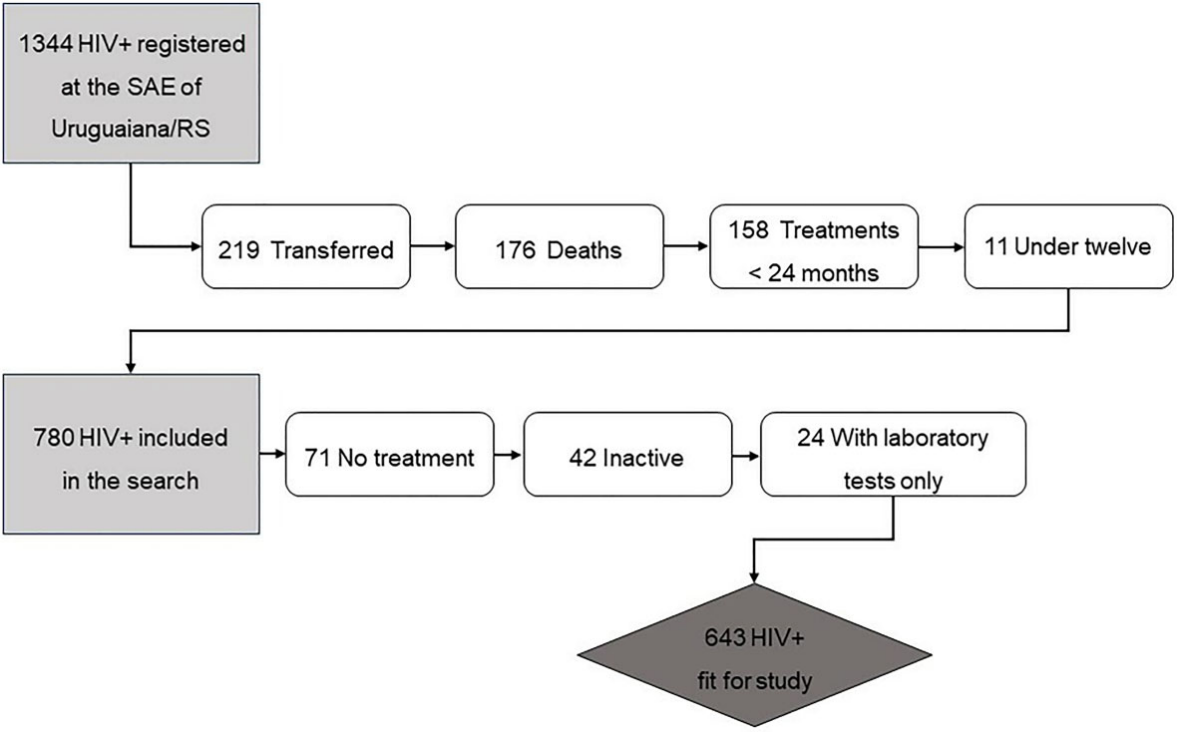
Flowchart of selection of patients for study participation. SAE: Specialized Assistance Service; RS: Rio Grande do Sul.

Of the 1,344 patients in the Specialized Assistance Service at the time of the study, 780 (58%) individuals met the criteria, and 137 (17.5%) were excluded because they did not start treatment, did not obtain the medication for at least 24 months, or had never acquired cART medication but had laboratory tests at some point. Therefore, 643 individuals participated in the study (Figure 1).

### Sociodemographic characteristics

Regarding the sociodemographic characteristics (Table 1), most participants were women (50.2%). The average age and standard deviation were 42±11.8 years, with a minimum of 12 and a maximum of 75 years of age. Ethnically, most participants considered themselves white (30.9%). Approximately 90% of the information on marital status and education was incomplete. Regarding the prescribed cART regimens, 20 different types of medications were dispensed. The most frequently prescribed cART regimens were Tenofovir + Lamivudine + Efavirenz, Atazanavir + Tenofovir + Lamivudine + Ritonavir, and Zidovudine + Lamivudine + Efavirenz with 38.7, 36, and 13.8%, respectively. Notably, 39.2% of the patients had already changed their initial treatment and 10.6% had, at some point, viral genotype testing.

**Table 1:** Characteristics of HIV-infected patients undergoing combination antiretroviral therapy (cART) registered at the SAE of Uruguaiana, RS.

Sociodemographic variables	n (%)
Gender	
Female	323 (50.2)
Male	320 (49.8)
Age (years)	
12-19	21 (3.3)
20-29	76 (11.8)
30-39	175 (27.2)
40-49	193 (30.0)
50-59	137 (21.3)
≥60	41 (6.4)
Antiretroviral treatment	
TDF + 3TC + EFZ	250 (38.9)
ATV + TDF + 3TC + RIT	232 (36.1)
AZT + 3TC + EFZ	91 (14.0)
Others	70 (11.0)
cART change	
Yes	252 (39.2)
No	391 (60.8)
Viral genotyping	
Yes	68 (10.6)
No	575 (89.4)
Viral load HIV-1 RNA (copies/mL)	
Undetectable	356 (55.4)
Detectable	187 (29.0)
No results	18 (2.8)
No exams	82 (12.8)
Over time change of viral load HIV-1 RNA	
Undetectable at all times	323 (50.2)
Lower	96 (14.9)
Higher	46 (7.2)
No exams or just a registered exam	178 (27.7)
CD4 + (cells/mm^3^)	
0-200	33 (5.1)
201-350	39 (6.1)
351-500	45 (7)
≥501	75 (11.7)
No results	369 (57.3)
No exams	82 (12.8)

SAE: Specialized Assistance Service; SD: standard deviation; TDF: tenofovir; 3TC: lamivudine; EFZ: efavirenz; ATV: atazanavir; RIT: ritonavir; AZT: zidovudine.

As for the HIV-1 RNA viral load, the analyses were performed using the results of tests carried out within the study period. In 50.2% of the studied subjects, the viral load remained undetectable, in 14.9%, it decreased in the first and last viral load measurements, and in only 7.2% was the viral load higher at the end of the study than in the beginning. Notably, 44.8% of HIV notifications performed via the Notifiable Diseases Information System (SINAN) were not found in the records.

### cART adherence and delay

Only 12.4% of the patients had 100% adherence in the 24-month period (i.e., obtained cART medication from the Specialized Assistance Center 24 times), followed by 42.5% of patients with minimally effective adherence, 20.8% of patients with low adherence, and 24.3% were non-adherent patients (Table 2). Despite the total number of delayed days, some patients were late by 1 day while others were behind by 544 days to restart the treatment. The average number of delayed days (over 34 days from the previous dispensation) was 18±29.6 days, with a minimum of 1 day and a maximum of 498 days. The average number of times patients were late to obtain the cART medication was 5.6±3.4 during the 24-month follow-up period, ranging from 1 to 16 times. Furthermore, 4.2% of PLWHA were never late for cART replacement, 37.6% were late less than 5 times, 47.4% were late 5 to 10 times, and 10.7% were late 11 to 16 times.

**Table 2:** Treatment adherence and delay of people living with HIV and AIDS in the municipality of Uruguaiana, RS.

Adherence level	n (%)
Adherent	80 (12.4)
Minimum effective adherence	273 (42.5)
Low adherence	134 (20.8)
No adherence	156 (24.3)
Delay	
1-4	242 (37.6)
5-10	305 (47.4)
11-16	27 (4.2)
No Delay	27 (4.2)

### cART adhesion and delay indicators

An estimate of 0.83 was found for the median value for adhesion indicator. Furthermore, values of 0.09 and 0.21 were found for delay in days and times the patient was late to replace treatment during the 24 months of follow-up, respectively (Table 3). For the adhesion indicator, the closer the value to 1, the better the population adherence, while for delay, the opposite is true (the closer the value to zero, the better). Analyses using Spearman's correlation (rho) and HIV-1 RNA viral loads were used to interpret these correlations. Significant statistical values (P≤0.01) and weak correlations (<0.4) were found between adherence and number of delayed days for cART replacement (rho=-0.349 and 0.198, respectively). The same analysis was performed to evaluate CD4+ cell counts, although no results were statistically significant.

**Table 3:** Assessment of combination antiretroviral therapy (cART) adherence and delay by epidemiological indicators.

Indicator	Median (25-75 percentiles)	Correlation with viral load (copies/mL)	
		Spearman's rho	P value
Adherence	0.83 (0.67-0.96)	-0.349	≤0.01
Total delayed days	0.09 (0.03-0.19)	0.198	≤0.01
Number of delays	0.21 (0.13-0.33)	0.027	0.536

### Multivariate analysis

A multivariate linear regression model was developed to analyze the factors influencing adherence and delay outcomes (Supplementary Table S1). The variables age, sex, cART change, and ≥1000HIV RNA copies/mL of blood were included in the final models. The predictors significantly associated with lower adherence were age between 20 and 49 years, male sex, not having changed cART, and ≥1000 HIV RNA copies/mL of blood (β=-0.125, -0.111, -0.093, -0.039, -0.037, and -0.001, respectively), demonstrating that such characteristics decrease the adherence indicator. For delay, the variables significantly associated were age between 40 and 49 years and ≥1000 HIV RNA copies/mL of blood (β=0.051 and 0.001, respectively), representing an increase in this indicator. Interestingly, our findings suggested that the probability of the patient being late to obtain their cART medication is considerably higher if the subject is 30-59 years old and male (Supplementary Table S1).

## Discussion

According to the sociodemographic profiles, the number of women infected with HIV (50.2%) was higher than that of men, which is different from numerous studies developed in Brazil that reported a smaller HIV female population that has stabilized at 30 to 40% (4,5,7,15). Therefore, the scenario in Uruguaiana differs from the national scenario, in which women are not a priority population group. Prevention interventions are aimed at HIV-positive pregnant women, and actions to prevent new infections mainly target males, as they are the most affected population group (16). Most participants were considered young adults since ∼80% were between 30 and 59 years old. Moreover, HIV is known to affect individuals of all age groups, and survival rates are directly associated to the individuals, medical care, and social factors (17). Our data corroborated research demonstrating that HIV is more prevalent in this age group in Brazil and other parts of the world, such as one study in India in which patients were aged between 39 and 50 years and another study in southern Brazil in which the average age of HIV-infected patients was between 30 and 39 years (5,18).

There is evidence that ethnicity may be associated with social and economic inequality, thus making clinical follow-up and treatment of seropositive non-white individuals challenging. This is a consequence of daily barriers and institutional racism, as confirmed by the increasing number of AIDS cases in the black population (19). In the last decade, AIDS cases have increased by 37.7% in self-reported brown people, while the increase in white individuals was 20% (4). In our study, 100 patients were considered brown/black, although more than half of the participants (344 people) lacked this information in the databases. Worryingly, our findings pointed to a lack of information on education and marital status. When data are not correctly or entirely submitted to the information system, the available data may not reliably reflect the reality of certain population aspects (15).

Additionally, a small number of patients lacked information in the SINAN system (44.8%). As this database is the official source of information used by the MH to generate national data on HIV/AIDS in children and adults (20), there are some fragilities in the epidemiological surveillance services. According to the Brazilian AIDS Epidemiological Bulletin of 2019, 70.6% of cases were reported in the SINAN from 2000 to June 2019. Among the unreported cases, 21.8% were found in the SISCEL/SICLOM systems and represented as under-reported SINAN cases (4). The constant lack of data poses difficulties for health professionals, which highlights the need for continuous education strategies to convey the real value of such information. Moreover, these data are pivotal for planned actions so that decisions can be made based on evidence, making it possible to achieve effective results against the AIDS epidemic (19).

Immediate initiation of cART is currently recommended for all PLWHA, regardless of their clinical and/or immunological status (8). Nonetheless, contraindications, adverse effects, drug resistance, and the Informative Note No. 03/2018 recommending the replacement of antiretroviral therapy regimens containing non-nucleoside reverse transcriptase inhibitors or protease inhibitors with dolutegravir in HIV-infected people with viral suppression over 12 years of age may lead to the replacement of cART with alternative regimens in this population (8,21). The 20 different therapeutic regimens found in this study were a consequence of this. According to the prevalence of primary or transmitted HIV resistance in the population, viral genotype testing is indicated due to its efficacy and cost-effectiveness. In 2015, the National Network for Drug Resistance Surveillance reported a 9.5% prevalence rate of primary mutations in protease inhibitors and reverse transcriptase (nucleotide analogs and non-analogs) (8). When considering nucleoside reverse transcriptase inhibitors alone, the national prevalence of mutations conferring resistance to this class was 5.8%, ranging from 4.5% in northern and northeastern Brazil to 7% in the south (7,22). This might explain the significantly low number of people in our study who performed this test (10.6%).

Moreover, according to laboratory tests, 50.2% of the subjects had undetectable HIV-1 RNA viral loads in the 24-month period. This finding is relevant because viral suppression results in a 96% lower risk of PLWHA to transmit the virus, thus preventing the development of resistant virus variants and improving the quality of life of these people (23). Maintaining an undetectable viral load is also important for reducing the risk of sexual HIV transmission, especially because heterosexual transmission is playing an increasingly important role in the HIV epidemic (4). This is evident in two large studies that showed that there was no case of HIV transmission from a person with a suppressed viral load to their respective seronegative partner, thus helping decrease the spread of the infection (24,25).

The initiation of treatment in patients with low LT-CD4 counts is essential, especially in patients with CD4 lymphocytes <200 cells/mm^3^, which leads to unnecessary morbidity and mortality rates, as it exposes the individual to the risk of opportunistic infections (26). In our study population, 5.1% were in this risk zone. The frequency with which CD4+ cell count tests are requested to monitor PLWHA depends on the clinical situation. Those with a CD4+ cell count below 350 cells/mm^3^ should have the test done every six months (8). In our study, this group accounted for 7% of the subjects. According to MH regulations, if the patient has two consecutive exams with a CD4+ cell count above 350 cell/mm^3^ in at least six months, the health professional does not need to order this test, but only the HIV-1 RNA viral load test. As a result of this regulation, 57.3% of the study subjects did not require a CD4+ cell count test, thus demonstrating a good immune profile in the population (8).

cART adherence was verified through cART medication acquisition records on the official platforms of the Brazilian government, and 12.4% were considered adherent because they continued treatment in all months of the follow-up period. Furthermore, 54.9% of the patients showed minimum effective adherence. This shows that our study presents a situation that is very distant from the 95% of adherent patients preconized by the Paris Charter Agreements (3).

Court et al. (27) demonstrated the importance of adherence and noted that the risk of virological failure decreased by 73% with every 10% increase in adherence measured using drugstore refill records. Adherence to cART is known to influence the risk of infection transmission, and early treatment initiation significantly reduces mortality and disease progression (24). In addition, non-adherence or discontinuation of cART favors the development of mutated viruses, making them drug-resistant (28).

Our study showed that the average delay in taking the cART was 18 days. Given that medications are provided for a 30-day treatment, this delay increases viral replication and compromises drug efficacy (10). Cruz et al. (29) reported that 80% of HIV-infected patients with undetectable HIV-1 RNA viral loads did not take longer than five days to seek cART acquisition. Delay in cART medication acquisition can be detected month by month during pharmaceutical care, and monitoring drug dispensation delay can be used to identify patients with cART adherence problems and prioritize these subjects in health service care.

Health indicators are used as tools to identify, monitor, and evaluate actions for patient care and assist in directing actions that can improve health practices. Monitoring medication dispensing through these indicators is a strategic point of care because it is closely related to the quality of the health service and treatment, and these indicator methods can be developed to bring health services closer to the patient and achieve successful therapy (30).

Martin et al. (9) showed that successful treatments must achieve a TCD4 cell count ≥200 cells/mm^3^, undetectable viral loads, and cART medication acquisition above 95%, as values below 95% are associated with a greater degree of virological failure and viral resistance. Low adherence was closely correlated to male sex, low education, and form of HIV transmission (31). Sangeda et al. (26) obtained 51.8% adherence while evaluating cART medications acquisition and found that old age, low alcohol consumption, and clinical staging were associated with good adherence. Gerenutti et al. (32) pointed out that successful therapy is more common in individuals who have access to pharmaceutical assistance, undetectable viral loads, and higher TCD4 + lymphocyte levels. Therefore, our adherence indicators could be applied to other HIV/AIDS specialized assistance services in Brazil. Besides that, this model could be evaluated and implemented for the treatment of hepatitis C virus, which is expensive and where treatment adherence also influences sustained virologic response.

The median value of the adhesion indicator was 0.83, which demonstrated that most subjects had a cART adherence above 80%, as recommended by the Clinical Protocols and Therapeutic Guidelines (8). These results do not indicate optimal adherence, as high levels of cART adherence are required for long-term benefits, including suppressing viral replication, delaying disease progression, and preventing the emergence of viral resistance (1). Moreover, this indicator showed that a considerable portion of the studied population was classified as low adherents or non-adherents to cART, and 25% of the subjects had an adhesion indicator value of 0.67 or less.

Both indicators of delay presented median values close to zero: 0.09 for total days delayed and 0.21 for number of delays. These two approaches are distinct ways of looking at the patient's profile of drug therapy, since the total number of days delayed measures the number of days after 34 days since the last dispensation before replacing the cART medication, and the number of delays shows how often the person was late in taking the drugs during the study period, regardless of the number of days delayed. These two measures allow a better understanding of patients with difficulties in adhering to cART, which is important because delays affect treatment.

Gomes et al. (33) studied patient compliance when starting cART and reported that only 11.8% of patients regularly obtained the medication, while 30.3% of patients were considered to have abandoned their treatment, and 57.9% obtained medication irregularly. TCD4^+^ lymphocyte counts above 200 cells/mm^3^ (P=0.006) and use of protease-free regimens (P=0.01) were recorded in patients with irregular medication acquisition and abandoned treatment. Pharmacy refill is a promising alternative method for verifying adherence focused on drug availability (14). Cruz et al. (34) reported a correlation between mean pharmacy visit intervals less than 33 days and <50 HIV RNA copies/mL (34). Cruz et al. (29) showed that PLWHA who missed cART medication acquisition for over 20 days often correlated with detectable viral load levels.

A statistically significant correlation between the two indicators (adhesion and total days delayed) and HIV-1 RNA viral load was observed (P≤0.01). However, Spearman's coefficients demonstrated a weak correlation. The negative coefficient value of the adhesion indicator (-0.349) means that adhesion is inversely proportional to viral load (the lower the adhesion, the higher the viral load values). These data are corroborated by other studies that stated that patients undergoing therapy have higher CD4+T lymphocyte levels and lower viral loads (32,35). Moreover, the positive value of Spearman's rho for total days delayed indicator (0.198) was directly proportional to viral load (the longer the delay in replacing the drug, the higher the viral load values), as also found in other studies (14,29).

Three linear multivariate models were developed to characterize the influence of each variable on the indicator outcomes previously mentioned, and it was found that some age groups and men never changed cART medication and >1000 HIV-1 RNA copies/mL, contributing negatively to the outcomes and posed risks to the success of the therapy. Risky adherence to treatment is significantly higher in adults than in people over 60 years of age because the latter are more mature and therefore more capable of taking care of their health (36). Additionally, men tend to neglect their health more than women, given they have worse health and do not make the necessary behavioral changes to prevent HIV transmission, which contributes to the risk of non-adherence and delay in treatment dispensation (35). The fact that not changing cART proved to be a factor compromising adherence is an alert for drug selection in patient treatment, since cART is a complex regimen that may be closely related to administration difficulties, side effects, and/or adverse effects, leading to lower adherence in some patients (17). The last variable contributing to poor cART adherence and dispensation delay was high HIV viral loads. The association was found in other studies that demonstrated that suboptimal adherence is strictly related to high viral loads and that the sum of low cART adherence and increased viral loads are associated with more than one day of missed doses of therapy (37,38).

The limitations of this study include the collection of secondary data on information systems, although they are official government systems used by the Brazilian health surveillance. In addition, the lack of information (∼90%) on education level, marital status, and ethnicity is of concern and did not allow these variables to be included in the multivariable model. The adherence assessment was verified indirectly from the dispensation of cART medication. Another limitation of the study was the lack of routine tests during the study period. Finally, two factors that may contribute to the discussion on adherence and that could not be included due to lack of data are use of other medications and time of patient diagnosis.

The predictive factors for adherence and delay (P≤0.01) that put the success of the therapy at risk were age between 20 and 59 years, male sex, not having changed the cART, and >1000 HIV RNA copies/mL in blood. This might be a simple and important tool in identifying patients who need assistance, providing them with the appropriate treatment, and focusing health service efforts on this population group because they lack cART adherence and have high morbidity and mortality risks. In addition, identifying factors associated with poor adherence and delays in cART medication acquisition helps characterize patients at a higher risk of unsuccessful therapy, allowing health professionals to systematize measures that favor adherence and prevent patients from abandoning therapy.

## Supplementary Material

Click to view [pdf].
